# Multifactorial control and treatment intensity of type-2 diabetes in primary care settings in Catalonia

**DOI:** 10.1186/1475-2840-9-14

**Published:** 2010-03-29

**Authors:** Lucas Mengual, Pilar Roura, Marta Serra, Montserrat Montasell, Gemma Prieto, Sandra Bonet

**Affiliations:** 1 Health Care Centre Badia del Vallès, Badia del Vallès, Barcelona, Spain; 2 Health Care Centre Ca n'Oriac, Sabadell, Barcelona, Spain; 3 Health Care Centre Rosa dels Vents, Barberà del Vallès, Barcelona, Spain; 4 Unitat de Suport a la Recerca, Ámbito Centro, Barcelona, Spain; 5 Adknoma Health Research, Barcelona, Spain

## Abstract

**Background:**

Many studies on diabetes have demonstrated that an intensive control of glycaemia and the main associated risk factors (hypertension, dislipidaemia, obesity and smoking) reduce cardiovascular morbi-mortality. Different scientific societies have proposed a multifactorial approach to type 2 diabetes.

The objective of this study was to identify the degree of control of glycosylated haemoglobin (HbA1c) and of cardiovascular risk factors in type 2 diabetic patients, using the GedapS 2004 guidelines, and to analyse the type and intensity of drug treatment.

**Methods:**

This cross-sectional, multicentre, epidemiological study was conducted in a primary care setting in Vallès Occidental South, Catalonia. Data were collected of 393 patients aged 18 and above who were diagnosed with diabetes mellitus type 2. Biodemographic and clinical data, cardiovascular risk factors, associated cardiovascular disease, and treatment were assessed. Descriptive and multivariable analysis with logistic regression was realized.

**Results:**

A total of 392 patients with a mean age of 66.8 years (SD = 10.6) (45.4% male patients) were analyzed. The duration of diabetes was 8.4 years (SD = 7.6). The degree of multifactorial control of risk factors was only 2.6%, although in more than 50% individual cardiovascular risk factor was controlled, except for LDL cholesterol (40.6%) and systolic blood pressure (29.6%). Furthermore, only 13.0% of subjects had an optimal BMI, 27.5% an optimal waist circumference. Treatment for diabetes was prescribed in 82.7% of patients, for hypertension 70.7%, for dyslipidaemia 47.2% and 40.1% were taking antiplatelets.

**Conclusion:**

Over 50% of type 2 diabetic patients presented optimal control of the majority of individual cardiovascular risk factors, although the degree of multifactorial control of diabetes was insufficient (2.6%) and should be improved. Drug treatment can be intensified using a larger number of combinations, particularly in patients with target organ damage and associated clinical cardiovascular disease.

## Background

Diabetes mellitus (DM) is a chronic disease with a prevalence of 4.5% - 18.5% in Spain [1]. According to the World Health Organisation (WHO), the prevalence of diabetes in Spain was expected to increase by as much as 40% between 2000 and 2025 [2]. DM is the seventh leading cause of death in Spain [3].

Several studies [4-7] in diabetic patients have shown that close control of glycaemia and the main cardiovascular risk factors, such as arterial hypertension (AHT), dyslipidaemia, obesity and smoking, reduces cardiovascular morbimortality.

Furthermore, the United Kingdom Prospective Diabetes Study (UKPDS) has shown that for every 1% reduction in glycosylated haemoglobin (HbA1c) there is a decrease in any DM-related complication and in mortality [8]. However, recent studies [9-11] recommend that HbA1c levels of 7% should be achieved and maintained in adult patients, without dropping below 6.5%.

Based on current evidences, different scientific societies have proposed using an approach using multifactorial control of risk factors in patients with type-2 diabetes (DM2)[12-14].

Despite these recommendations [14,15], multifactorial control of risk factors in diabetes is still insufficient [16-18]. Thus, for example, Mostaza et al [19], reported that optimal control of all risk factors was found in just 7% of diabetic patients. The current consensus is that diabetic patients should be seen at a primary care centre for the purpose of prevention and control [20], and this is also ratified by the WHO document [21]. In this context, our study was aimed to identify the degree of control of HbA1c and multifactorial control of risk factors in type 2 diabetic patients, using the GedapS 2004 guidelines [22].

## Methods

### Study design and population

The study had a cross-sectional, multicentre, epidemiological design. It focused on primary care in normal clinical practice provided in the Vallès Occidental South region in the north of the province of Barcelona. The region had a population of 429,816 persons. Forty physicians were selected using simple randomisation from a total of 186 physicians belonging to 16 primary care teams in 8 towns in the zone. The physicians who agreed to participate in the study were given a specific training session to explain the study objectives, procedures, and, in particular, how to collect data and record it in the Case Report Form (CRF). The study was approved by the Institutional Review Board of the Jordi Gol Institute for Research in Primary Care (Institut d'Investigació en Atenció Primària Jordi Gol; IDIAP Jordi Gol).

The patients were recruited according the following selection criteria:

#### Inclusion criteria

patients aged 18 and above diagnosed of DM2 at least 6 months prior to study inclusion and who had given informed consent to participate in the study.

#### Exclusion criteria

patients with type 1 diabetes mellitus (DM1), patients with DM2 in a terminal phase and those with a severely deteriorated quality of life or who would have had difficulty in attending the centre during the study period, and patients who, in the investigator's opinion, presented any condition which could hinder their participation (communication problems, cognitive or sensorial disorder, language barrier and severe psychiatric disorders).

The sample size was calculated in terms of the primary objective (to determine the degree of control of HbA1c in patients with type-2 diabetes attended at primary care in Vallès Occidental South, using the GedapS 2004 guidelines). A previous study (*TranSTAR study*) [17], found that HbA1c was controlled in 18.8% of patients. Using the binomial distribution, 390 patients with type-2 diabetes would provide an accuracy of 4% for estimating the proportion of diabetic patients with good HbA1c control, with a 95% confidence interval, assuming that 10% of patients would not be valid for the analysis.

### Data collection

Between July 2007 and January 2008, each physician included the first diabetic patient attending his/her primary care practice who met the inclusion criteria, up to a total of 10 patients per physician in the course of 10 days.

The following study variables were recorded in an electronic CRF, applying internal consistency rules to ensure data quality control: biodemographic data (age, sex, weight, height, waist circumference, year of diagnose with DM2), physical exercise (hours/week), cardiovascular risk factors, associated cardiovascular disease, smoking habit, clinical data (blood pressure, heart rate, blood tests, kidney function, proteinuria, glycaemic and lipid profile) and treatments (antidiabetics, antihypertensives, antidyslipidaemics, antiplatelets, anticoagulants).

### Measurement and diagnostic criteria

#### Normal values

We used the normal values recommended in the European Guidelines [23] for blood pressure (BP), obesity, sedentary lifestyle, smoking and alcohol.

#### Blood pressure (BP)

It was measured as recommended by the ESH-ESC guidelines [24]. BP was recorded twice per visit and the mean value calculated.

#### Waist circumference

It was measured in centimetres with a tape measure at the midpoint between the lower part of the last rib and the top of the iliac crest.

#### Left ventricular hypertrophy

It was assessed following ESH-ESC guidelines [24]: Sokolow criteria (SV1+RV5-6>38 mm), Cornell criteria (RaVL+SV3>28 in men and 20 in women) or by echocardiography.

#### Body Mass Index (BMI)

The BMI is used to classify a person's weight status. It is calculated using the formula: weight (kg)/height (m^2^).

#### Dyslipidaemia

It was diagnosed following the criteria set out in the clinical guidelines [25], which consider to be indicative of hypercholesterolaemia a total cholesterol level of ≥ 200 mg/dL in primary prevention, or an LDL-cholesterol level of ≥ 100 mg/dL in secondary prevention.

#### Cardiovascular Risk

It was calculated using Framingham Risk Tables [26] which estimate the 10-year risk of suffering a coronary event, angina or fatal or non-fatal myocardial infarction. Low cardiovascular risk was considered as less than 5%, slight risk as 5-9%, moderate risk as 10-19%, high risk as 20-39% and very high risk as over 39%.

### Objective

Study's primary objective was to determine the degree of control of HbA1c and multifactorial control of risk factors in type 2 diabetic patients, using the GedapS 2004 guidelines [22]. This guideline consider good control as fulfilment of the following conditions: HbA1c lower than 7%, total cholesterol lower than 200 mg/dL, LDL cholesterol lower than 100 mg/dL, HDL cholesterol higher than 40 mg/dL, triglycerides (TG) lower than 150 mg/dL, systolic blood pressure (SBP) lower than 130 mmHg, diastolic blood pressure (DBP) lower than 80 mmHg and not smoking. When all these conditions are fulfilled simultaneously, it can be considered that a patient has good multifactorial control. This guideline also recommends a body mass index (BMI) between 18.5 and 24.9 kg/m^2 ^and a waist circumference of less than 102 cm for men and 88 cm for women.

### Statistical methodology

All analyses were performed on a single sample of diabetic patients. Evaluable patients were all those who met the selection criteria and had a recorded value of the principal study variable (HbA1c). The qualitative variables were described using absolute and relative frequencies, whereas the quantitative variables were described using their mean, standard deviation, median, minimum and maximum, including the total number of valid values. Parametric tests (Student's t or ANOVA) or non-parametric tests (Mann-Whitney or Kruskal-Wallis) were used to compare quantitative variables for patient subgroups, depending on the characteristics of the variable being studied. The chi-squared test was used for qualitative variables. Logistic regression was performed to assess the association between good DM2 control (HbA1c) and the independent variables found to be of interest in the bivariate analysis. All statistical analyses were performed with a two-tailed confidence level of 95%. The SAS statistical package was used (version 9.1.3).

## Results

### Clinical and analytical characteristics

A total of 393 patients with DM2 were recruited, although one was subsequently excluded as the principal study variable was not specified, to give a final total of 392 patients. The mean age was 66.8 years (SD = 10.6). 54.6% were women.

The mean duration of DM2 was 8.4 years (SD = 7.6). 44.8% of patients were obese (BMI ≥ 30). The mean BMI was 29.3 kg/m^2 ^in men and 30.6 kg/m^2 ^in women (p < 0.01). Waist circumference was higher than recommended (≥ 102 cm in men and ≥ 88 cm in women) in 51.4% of the men and 83.1% of the women; this difference was statistically significant (p < 0.05). A total of 11.1% of the patients were active smokers, 81.3% of whom were male (44.2% ≤ 60 years of age).

As regards physical activity, more than half the sample (59.0%) undertook regular physical exercise (walking as a minimum) for about one hour an average of 5 days per week.

**Figure 1:**
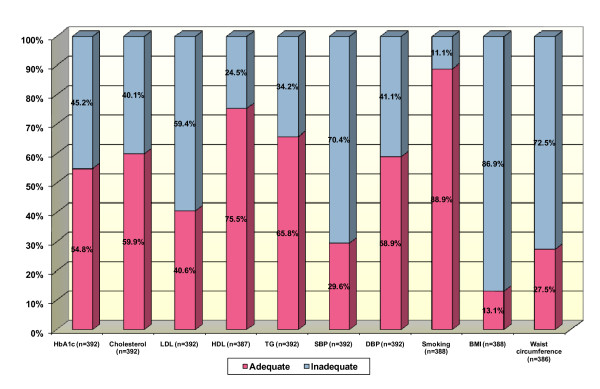
**Degree of cardiovascular risk factor control according to GedapS 2004 in the DM2VALLES study population**. Adequate control: Hb1Ac < 7%; Cholesterol < 200 mg/dl; LDL < 100 mg/dl; HDL > 40 mg/dl; TG < 150 mg/dl; SBP/DBP < 130/80 mmHg; BMI: 18.5-249 kg/m2; Waist circumference < 102 cm in men and < 88 cm in women.

When we analysed the degree of control of cardiovascular risk factors, individually, the majority had good control of over 50%. It should be noted that 54.8% of the sample had a HbA1c value of less than 7%. Factors that had poor control included SBP < 130 mmHg (29.6%), LDL cholesterol < 100 mg/dL (40.6%), BMI < 25 (13.1%) and waist circumference < 102 cm in men and 88 cm in women (27.5%) (Figure 1). The most relevant clinical characteristics are listed in Additional file 1. Good BP control (SBP < 130 and DBP < 80) was found in 24.7% of the sample. All cardiovascular risk factors were well controlled in just 2.6% of the diabetic patients in this study, using the criteria set out in GedapS 2004.

### Cardiovascular Risk: Cardiovascular risk factors and associated complications

Cardiovascular risk (CVR), as determined using the Framingham Risk Tables, showed that 23.2% of patients presented a low risk, 37.5% a slight risk, 30.1% a moderate risk, 8.4% a high risk and 0.8% a very high risk.

The most prevalent cardiovascular risk factors were AHT (73.0%), dyslipidaemia (58.2%) and obesity (44.8%). Microvascular complications were present in 26.0% of patients, the most common being nephropathy (17.8%; (13.5% with microalbuminuria and 3.7% with proteinuria)) and retinopathy (12.1%).

Macrovascular complications were present in 24.5% of the sample, with ischaemic cardiopathy (12.5%) and peripheral arteriopathy (11.0%) being the most prevalent. In all, 44.9% of the sample had some type of target organ damage and/or clinical cardiovascular disease.

### Factors associated with control of HbA1c

In the bivariate analysis, factors associated with a good control of HbA1c (<7%) to a statistically significant degree (p < 0.05) were: shorter history of DM2; female patients; lower SBP level; lower triglycerides (TG) level; lower albumin level in urine; higher LDL cholesterol; less intensive treatment for DM2, dyslipidaemia and antiplatelets; and lower CVR (Additional file 2).

The logistic regression analysis showed that the probability of good HbA1c control (<7%) was 4.09-fold higher in patients taking one or two antidiabetic drugs compared with those taking more than two antidiabetic drugs. Furthermore, this probability was 2.62-fold higher in patients with no retinopathy than in those with retinopathy.

### Drug treatment description

82.7% of patients in this study took antidiabetic drugs. 52.8% of them were on monotherapy (59.2% on metformin) and 47.2% took a combination therapy. 14.9% of patients had insulin treatment either alone or in combination with oral drugs. Likewise, 70.7% of patients took antihypertensives (93.4% of patients diagnosed with AHT), either in monotherapy (42.2%) or combination therapy (57.8%). 47.2% were being treated for dyslipidaemia (75.9% of patients diagnosed with dyslipidaemia), 40.1% were taking antiplatelet drugs and 5.6% anticoagulants (Additional 3). As regards the cardiovascular risk, 16.7% of patients considered to be at high or very high risk (≥ 20) were not taking any treatment for DM2, 22.2% were not on antihypertensives, 52.8% were not receiving treatment for dyslipidaemia and 55.6% were not on antiplatelets.

With regard to treatment intensity and the presence of target organ damage and/or clinical cardiovascular disease, there was significantly higher treatment intensity for hypertension and anti-platelets. There were no differences in treatment intensity for diabetes and dyslipidaemia (Additional file 4).

Of the patients with HbA1c ≥ 7%, 31.2% were being treated with one oral drug and 64.6% were on combination therapy, 39.4% of whom were taking two oral drugs, 2.9% taking three, 18.2% one oral drug plus insulin and 4.2% insulin combinations. Finally, 20.7% of patients presented kidney disease, 72.8% of whom were taking angiotensin converting enzyme inhibitors (ACEI) or angiotensin II receptor blockers (ARB).

## Discussion

The primary objective of the DM2VALLES study was to identify the degree control of HbA1c, the degree of individual and multifactorial control of risk factors in type 2 diabetic patients attended at primary care centres. According to other studies [19,27] we only found good simultaneous control of all risk factors in 2.6% of the study population, although there was good control of individual factors such as HbA1c, total cholesterol, HDL cholesterol, TG, DBP and smoking habit. The hardest parameters to control were SBP and LDL cholesterol, as also reported previously [27,28].

The degree control of HbA1c was found to be related to the duration of diabetes. This control worsened with length of illness due to the progressive deterioration of the beta cells, as reported by other authors [4,5,18]. The high percentage of patients with good HbA1c control found in our study contrasts the values obtained in other studies, despite their similar duration of diabetes (8.4 years) [18]. This may be explained by the health policy that has been implemented in this area for several years on intervention and control in diabetes.

One important finding which should be highlighted is the high number of patients with a BMI of ≥ 30 kg/m2 (44.8%), a figure which is similar to those found in previous studies [18,29,30] and which also contributes to the poor multifactorial control of our patients. Indeed, weight loss can result in as much as 0.5 reductions in the HbA1c percentage [12] since a reduction in body fat is associated with reduced insulin resistance and therefore better glycaemic control.

The benefits of regular physical exercise in the general population, and in diabetic patients in particular, are well known. Indeed, exercise is a key component in the treatment of diabetes, together with diet, as it also helps to improve cardiovascular risk factors [12,13]. Our study showed positive results in this area, because over half the study population exercised on a regular basis.

The number of smokers in our sample was lower than in other studies, although the prevalence of smoking in diabetic male subjects under the age of 60 was higher than in the general population in Catalonia [16]. Controlling this risk factor should be prioritized in diabetics in light of the relationship between smoking and micro- and macrovascular complications and mortality [13,22].

The prevalence of nephropathy, retinopathy, ischaemic cardiopathy, stroke and peripheral arteriopathy observed in our study was similar to that observed in other studies carried out recently in Spain [18,29].

Although patients with DM2 are characterised by their high level of cardiovascular risk, our results based on the Framingham Risk Tables showed that, in contrast to previous studies [28,29,31], the majority of the sample (60.7%) had a low or slight risk (<10%). These findings are a result of the good individual control of the majority of the cardiovascular risk factors presented by the patients in our study.

As regards treatment, we found that the majority of patients were taking antidiabetics, with good control of HbA1c levels in more than 50% of the sample. However, we found a low use (15.6%) of insulin in monotherapy or combination therapy. The number of patients receiving insulin treatment varies considerably from one study to another. Thus, the percentage is as low as 9.6% in some cases, and in others it is almost twice higher (approximately 30%) [18,27,28].

An adequate control of LDL cholesterol level (<100 mg/dL) was observed in 40.6% of patients, which is notably higher than the low values reported in other studies also conducted in primary care settings [27,28]. Despite the findings of studies such as ATP III [32] and CARDS [33] which recommend pharmacological treatment, only 47.2% of our patients were on treatment for dyslipidaemia. The use of a more intensive treatment than hypolipidaemic drugs in diabetic patients should therefore be considered [13].

Although 70.7% of the sample was on antihypertensive treatment, only 24.7% presented good blood pressure control (<130/80 mmHg). In our study, 57.8% of patients were taking combinations of antihypertensive drugs. The review by Bakris et al.[34] showed that combination therapy with two or more drugs is required for good BP control in diabetic patients, which suggests that the patients in our study were being inappropriately treated in this respect and their treatment should be intensified by either increasing the dose or using combinations of drugs. A quarter of diabetic patients with kidney disease were not being treated with either ACEIs or ARB despite the fact this practice is recommended in the literature because these drugs have been shown to slow advancing kidney damage [35].

Antiplatelet treatment was being used in 40.1% of all patients, a similar value to that reported in other studies [16,27] and it was being used by 44.4% of patients in the high cardiovascular risk group. Indication for this treatment in primary prevention is currently controversial [36].

In the group of patients with high cardiovascular risk (≥ 20), where intensified treatment is particularly indicated, a high percentage were either receiving no drug treatment (16.7% for DM2, 22.2% for AHT, 52.8% for dyslipidaemia, 55.6% for antiplatelet agents) or only monotherapy (38.9% for DM2 and 36.1% for AHT). Treatment should also be intensified in patients with target organ damage and/or clinical cardiovascular disease, because many patients in these groups are not on treatment, or they are on monotherapy.

With regard to the limitations of our study, it should first be noted that the study population was recruited from a metropolitan area of Barcelona, which limits its validity to urban areas. Second, the study sample was chosen from a population seeking primary care. This means that we were unable to analyse the characteristics of diabetic patients who do not attend such primary care setting, although it the centres participating in this study have all been operating for more than 10 years and they cover more than 90% of the diabetic population in the region.

## Conclusions

The results of this study suggest that there is still room for improvement in the clinical and therapeutic management of patients with type-2 diabetes attended at primary care centres in our area. The purpose of this would be to achieve good multifactorial control of all risk factors, because previous studies [4,5] have shown that this strategy is beneficial in slowing down or preventing the appearance of chronic complications of type-2 diabetes. The elevated level of obesity that we observed in our study suggests that we should strengthen populational strategies aimed at improving the healthy lifestyle of the population.

## Competing interests

The authors declare that they have no competing interests.

## Authors' contributions

All authors participated in the design and the coordination of the study, reviewed the statistical analysis and participated in writing the manuscript. They all read and approved the final manuscript.

## Supplementary Material

Additional file 1 **Clinical and analytical characteristics in the DM2VALLES study population**. SD: Standard deviation, BMI: Body mass index; SBP: Systolic blood pressure; DBP: Diastolic blood pressure. Click here for file

Additional file 2 **Factors associated with glycosylated haemoglobin control in the DM2VALLES study population**. ^1^Chi-squared test; ^2^Mann-Whitney U test; BMI: Body mass index; HbA1c: Glycosylated haemoglobin; SBP: Systolic blood pressure; DBP: Diastolic blood pressure; CVR: Cardiovascular risk; DM2: Diabetes mellitus type 2. Click here for file

Additional file 3 **Treatment for the cardiovascular risk factors in the DM2VALLES study population**. ASA: Acetylsalicylic acid; ARB: Angiotensin-II receptor blockers; ACEI: Angiotensin converting enzyme inhibitors. Click here for file

Additional file 4 **Treatment intensity and the presence of target organ damage and/or clinical cardiovascular disease in the DM2VALLES study population**. ^1^Chi-squared test; DM: Diabetes Mellitus; AHT: Arterial hypertension; TOD + CCD: target organ damage and/or clinical cardiovascular disease. Click here for file
