# Supplementary material for: Multifactorial control and treatment intensity of type-2 diabetes in primary care settings in Catalonia
Source: Cardiovasc Diabetol. 2010 Mar 29;9:14. doi: 10.1186/1475-2840-9-14 (PMC2858123; doi:10.1186/1475-2840-9-14)
Supplement: Additional file 1 — Clinical and analytical characteristics in the DM2VALLES study population. SD: Standard deviation, BMI: Body mass index; SBP: Systolic blood pressure; DBP: Diastolic blood pressure. [file 1475-2840-9-14-S1.PDF]

Table 1. Clinical and analytical characteristics in the DM2VALLES study population.

**Total (n, %)**	**392**	**100.0**
**Sex (n, %)**		
Man	**178**	**45.4**
Woman	**214**	**54.6**
**Age, years (mean, SD)**	**66.8**	**10.6**
**BMI, Kg/m2 (mean, SD)**	**30.0**	**5.0**
**Waist circumference, cm (mean, SD)**	**102.2**	**12.9**
**Duration of diabetes, years (mean, SD)**	**8.4**	**7.6**
**Smoking habit (n, %)**	**388**	**100.0**
Smoker	43	11.1
Ex-smoker > 1 year	117	30.2
Never smoked	228	58.8
**SBP, mmHg (mean, SD)**	**137.8**	**16.1**
**DBP, mmHg (mean, SD)**	**77.0**	**9.8**
**Glycosylated haemoglobin % (mean, SD)**	**7.1**	**1.3**
**Cholesterol total, mg/dL (mean, SD)**	**191.9**	**37.8**
**HDL Cholesterol, mg/dL (mean, SD)**	**52.8**	**18.2**
**LDL Cholesterol, mg/dL (mean, SD)**	**111.8**	**34.9**
**Triglycerides, mg/dL (mean, SD)**	**142.6**	**86.0**
**Creatinine, mg/dL (mean, SD)**	**0.9**	**0.3**
**Albuminuria, µg/mg (mean, SD)**	**38.0**	**86.7**
