# Supplementary material for: Multifactorial control and treatment intensity of type-2 diabetes in primary care settings in Catalonia
Source: Cardiovasc Diabetol. 2010 Mar 29;9:14. doi: 10.1186/1475-2840-9-14 (PMC2858123; doi:10.1186/1475-2840-9-14)
Supplement: Additional file 2 — Factors associated with glycosylated haemoglobin control in the DM2VALLES study population. 1Chi-squared test; 2Mann-Whitney U test; BMI: Body mass index; HbA1c: Glycosylated haemoglobin; SBP: Systolic blood pressure; DBP: Diastolic blood pressure; CVR: Cardiovascular risk; DM2: Diabetes mellitus type 2. [file 1475-2840-9-14-S2.PDF]

Table 2. Factors associated with glycosylated haemoglobin control in the DM2VALLES study population

	HbA1c < 7%	HbA1c ≥ 7%	p[1,2]
**Female (%)**	59.1	49.2	0.049
**BMI ≥ 30 (%)**	46.0	43.4	0.611
**Smokers (%)**	10.7	11.3	0.849
**Antidiabetic treatment (%)**			
Monotherapy	72.1	35.3	<0.0001
Combination therapy	27.9	64.7	
**Antihypertensive treatment (%)**			
Monotherapy	43.4	40.8	0.660
Combination therapy	56.6	59.2	
**Dyslipidaemia treatment (%)**	42.3	53.1	0.033
**Antiplatelet treatment (%)**	34.9	46.3	0.021
**Age (mean, SD)**	66.8 (10.7)	66.8 (10.4)	0.889
**Waist Circumference (cm) (mean, SD)**	101.3 (12.6)	103.3 (13.2)	0.096
**LDL cholesterol (mg/dL) (mean, SD)**	110.4 (35.0)	102.0 (44.6)	0.049
**Triglycerides (mg/dL) (mean, SD)**	123.2 (63.1)	154.6 (113.0)	0.001
**Albuminuria (μg/mg) (mean, SD)**	25.0 (60.0)	54.2 (109.5)	0.000
**SBP (mmHg) (mean, SD)**	135.8 (15.1)	140.1 (17.0)	0.010
**DBP (mmHg) (mean, SD)**	76.5 (9.6)	77.7 (10.0)	0.152

**CVR (Framingham) (mean, SD)**	9.6 (7.0)	11.4 (8.0)	0.010
**Duration of DM2 (mean, SD)**	7.3 (7.6)	9.8 (7.5)	<0.000
