# Supplementary material for: Multifactorial control and treatment intensity of type-2 diabetes in primary care settings in Catalonia
Source: Cardiovasc Diabetol. 2010 Mar 29;9:14. doi: 10.1186/1475-2840-9-14 (PMC2858123; doi:10.1186/1475-2840-9-14)
Supplement: Additional file 3 — Treatment for the cardiovascular risk factors in the DM2VALLES study population. ASA: Acetylsalicylic acid; ARB: Angiotensin-II receptor blockers; ACEI: Angiotensin converting enzyme inhibitors. [file 1475-2840-9-14-S3.PDF]

Table 3. Treatment for cardiovascular risk factors in the DM2VALLES study population.

	n	%
**Antidiabetics**	**324**	**82.7**
Sulfonylureas	153	39.0
α-Glucosidase inhibitors	6	1.5
Metformin	232	59.2
Glitazones	27	6.9
Glinide	10	2.6
Total insulin	61	15.6
**Antihypertensives**	**277**	**70.7**
Diuretics	149	53.8
Calcium channel blockers	72	26.0
Beta-blockers	48	17.3
ACEI	150	54.2
ARA-II	79	28.5
Alpha-blockers	19	6.9
**Antidyslipidaemic agents**	**185**	**47.2**
Statins	164	88.6
Fibrates	24	13.0
**Antiplatelet agents**	**157**	**40.1**
ASA	133	33.9
Clopidogrel	22	5.6
**Anticoagulants**	**22**	**5.6**
