# Supplementary material for: Multifactorial control and treatment intensity of type-2 diabetes in primary care settings in Catalonia
Source: Cardiovasc Diabetol. 2010 Mar 29;9:14. doi: 10.1186/1475-2840-9-14 (PMC2858123; doi:10.1186/1475-2840-9-14)
Supplement: Additional file 4 — Treatment intensity and the presence of target organ damage and/or clinical cardiovascular disease in the DM2VALLES study population. 1Chi-squared test; DM: Diabetes Mellitus; AHT: Arterial hypertension; TOD + CCD: target organ damage and/or clinical cardiovascular disease. [file 1475-2840-9-14-S4.PDF]

Table 4. Treatment intensity and the presence of target organ damage and/or clinical cardiovascular disease in the DM2VALLES study population

	Without TOD + CCD		With TOD + CCD		p[1]
	n	%	n	%	
**Total of patients**	**215**	**100.0**	**176**	**100.0**	
**Without treatment for DM**	**57**	**26.4**	**28**	**15.9**	
**Monotherapy for DM**	104	48.1	96	54.5	**0.085**
**Combination therapy for DM**	55	25.5	52	29.6	
**Without treatment for AHT**	**75**	**34.9**	**38**	**21.6**	
**Monotherapy for AHT**	73	34.0	45	25.6	**0.000**
**Combination therapy for AHT**	67	31.1	93	52.8	
**Without treatment for dyslipidaemia**	**118**	**54.6**	**88**	**50.0**	**0.284**
**With treatment for dyslipidaemia**	**98**	**45.4**	**88**	**40.0**	
**Without antiplatelets treatment**	**164**	**75.9**	**71**	**40.3**	**0.000**
**With antiplatelets treatment**	**52**	**24.1**	**105**	**59.7**	
